# How safe is teaching radical cystectomy? Results from the prospective Swiss society of urology database

**DOI:** 10.1007/s00345-025-06173-4

**Published:** 2026-01-28

**Authors:** Matteo Scherrer, Lujza Brunaiova, Marc Furrer, Hubert John, Julien Schwartz, Ilaria Lucca, Philippe Sèbe, Agostino Mattei, Daniel Engeler, Räto T. Strebel, Stephen Wyler, Thomas RW Hermann, Daniel Nguyen, Michael Müntener, Beat Roth, Laila Schneidewind

**Affiliations:** 1 Department of Urology, University Hospital Bern, Inselspital, University of Bern, Wilhelm-Fabry-Haus, Freiburgstr. 37, CH-3010 Bern, Switzerland; 2 Department of Urology, Canton Hospital Winterthur, Winterthur, Switzerland; 3 Department of Urology, Hospital Cecile Lausanne, Lausanne, Switzerland; 4 Department of Urology, CHUV Lausanne, Lausanne, Switzerland; 5 Department of Urology, University Hospital Genf, Genf, Switzerland; 6 Department of Urology, University and Teaching Hospital of Lucerne, University of Lucerne, Lucerne, Switzerland; 7 Department of Urology, Canton Hospital St. Gallen, St. Gallen, Switzerland; 8 Department of Urology, Canton Hospital St. Graubünden, Chur, Switzerland; 9 Department of Urology, Canton Hospital St. Aarau, Aarau, Switzerland; 10 Department of Urology, Canton Hospital Frauenfeld, Frauenfeld, Switzerland; 11 Division of Urology, Department of Surgical Sciences, Stellenbosch University, Stellenbosch, Western Cape South Africa; 12 Hannover Medical School, Hannover, Germany; 13 Department of Urology, Hospital Neuchâtel – Pourtalès, Neuchatel, Switzerland; 14 Department of Urology, Hospital Zürich Triemli, Zürich, Switzerland

**Keywords:** Bladder cancer, Radical cystectomy, RARC, Surgical education, Surgical safety

## Abstract

**Purpose:**

To provide a detailed description of the Swiss Society of Urology prospective database of radical cystectomy (RC) due to bladder cancer (BC) about differences in operative outcomes between teaching and non-teaching RC.

**Methods:**

We collected the data of all RCs for BC from the register from March 2017 to March 2025, leading to 1587 cases. Afterwards, we decided about the extraction of 34 variables, e.g. operating approach and intraoperative complications. Furthermore, we determined that only complete data sets for our pre-defined variables will be included in the analysis, which led to 1304 RC cases.

**Results:**

Median patient age was 72.0 years (IQR 64.0–78.0). The majority of patients underwent open RC (*n* = 838; 64.3%). More than one quarter (*n* = 344; 26.4%) were teaching surgeries and there was no significant difference between both groups regarding demographic characteristics. There were no significant differences between teaching and non-teaching operative results in terms of surgical resection (R1 status; *p* = 0.295), duration of inpatient treatment (*p* = 0.394), infection (*p* = 0.023), wound healing disorders (*p* = 0.484), duration of surgery (*p* = 0.365), intraoperative bleeding (*p* = 0.635) and intraoperative blood loss (*p* = 0.074). However, in terms of the number of resected lymph nodes, blood transfusion rate, number of transfused packed red blood cells, intraoperative complications as well as the highest grade of complication during inpatient treatment teaching RC showed less favorable results, e.g. number of evaluated lymph nodes (teaching median 18.0 versus non-teaching median 20.0, *p* < 0.001).

**Conclusion:**

Teaching RC is safe, for a high complex procedure, according to our prospective pilot study.

**Supplementary Information:**

The online version contains supplementary material available at 10.1007/s00345-025-06173-4.

## Introduction

Radical cystectomy (RC) with urinary diversion is considered the standard treatment for muscle-invasive and selected high-risk non-muscle invasive bladder cancer (BC) [1, 2]. As one of the technically most complex urological surgeries, RC carries a considerable perioperative risk, technical demands and short- as well as long-term morbidity, even in experienced hands [3, 4, 5]. Whether performed open (ORC) or robot-assisted laparoscopically (RARC), the surgeon’s experience appears to influence the outcomes, including complication rates, oncological quality and overall patient recovery [6, 7, 8, 9]. Over the past decades, robot-assisted techniques have become more common, with the hope of minimizing blood loss, shortening hospital stays and reducing complications [3, 10]. However, these benefits are not consistent across all hospitals or surgeons, and the learning curve associated with RARC may be long and variable. While some studies show improvements after 20 to 30 cases, others find no clear plateau, especially for more complex outcomes such as recurrence rates [4, 6, 11]. The European Association of Urology (EAU) and also the American Urological Association (AUA) emphasize the role of experience and case selection in achieving the optimal outcomes for RC [1, 2]. Recent studies also highlight the impact of surgical experience as a critical independent factor for the outcomes of patients undergoing RC [6, 8, 10]. However, some research also indicates that patient factors and institutional volume may outweigh the influence of individual surgeon experience alone [7, 11]. Additionally, while most of those studies report learning curves and guidelines call for action to perform RC only in experienced, high-volume centers, one essential question remains: Is it safe to teach young surgeons in a complex procedure like RC?

A national cancer database is an important tool to explore trends in cancer care, to serve as the basis for quality improvement and to monitor education of surgical skills. Mandatory under the Highly Specialized Medicine (HSM) program in Switzerland to monitor the quality of BC care, the Swiss Society of Urology (SSU) has developed a prospective, nationwide registry to collect pre- and postoperative data of patients undergoing RC for BC. The collection of quality parameters on a large, multi-center scale can provide valuable data to all urologists on BC care and to ensure surgical improvement. Moreover, it may help those responsible for training to measure their own treatment accuracy and to compare anonymously the surgical quality.

Consequently, we performed a detailed pilot analysis from the SSU registry database with the primary aim to describe differences in operative results between teaching and non-teaching RC. We further performed a subgroup analysis for RARC, since it might become the gold standard for RC in the near future.

## Materials and methods

### Development of the study and study population

The study was designed according to the guidelines in the synthesis of qualitative research (ENTREQ) found on the equatornetwork.org, an international initiative providing robust reporting guidelines [12].

Participation to the SSU RC register under the nationwide HSM program is mandatory to be reimbursed for the RC procedures. Regular independent data audits are performed to ensure excellent data quality. Data (more than 80 elements about tumor stage, operation type or 30-day complications) are collected prospectively and anonymously via an electronic database. All procedures performed in this study were in accordance with the ethical standards of the institutional and/or national research committee and with the 1964 Helsinki Declaration and its later amendments or comparable ethical standards. A formal oral consent is required. The protocol was approved by the Cantonal Ethics Committee of Zurich (BASEC-Nr. Req-2017-00283).

For this analysis, we collected the data of all RCs for BC from the register from March 2017 to March 2025, leading to 1587 cases. Afterwards, we decided about the extraction of 34 variables (Supplementary), like operative approach, duration of surgery, blood loss, intraoperative complications, complications during inpatient treatment or number of evaluated lymph nodes, regarding our primary and secondary study aims. Furthermore, we determined that only complete data sets for our pre-defined variables will be included in the analysis, which led to 1304 RC cases. Lastly, we defined that Clavien-Dindo classification I and II will account as mild and III, IV as well as V as serious complications.

### Statistical analysis

For each numerical variable, the numerical distribution was preliminarily assessed by the Kolmogorov–Smirnov test. Descriptive statistics were made with means and standard deviations for normal distribution or with medians and IQRs for non-parametric data. For parametric continuous variables, Student’s t test was used, and for parametric categorical variables, the chi-square test or the Fisher exact test was used. For non-parametric data, the Mann–Whitney U test was used for categorical and continuous variables. All reported p-values were based on a two-sided hypothesis, and *p* < 0.05 was considered to be significant. All statistical calculations were performed using Statistical Package for the Social Sciences 29.0 software (SPSS Inc., IBM Corp., Armonk, NY, USA).

## Results

### Demographic characterization of the study population

Overall, the complete data sets of 1304 patients were included in the analysis. Median patient age was 72.0 years (IQR 64.0–78.0). As expected, the majority of the patients were male (*n* = 997; 76.5%). 838 patients (64.3%) underwent ORC. Table 1 illustrates the detailed demographic characterization of the whole study population. A detailed demographic characterization between teaching and non-teaching procedures is given in the Supplementary.

**Table 1:** Demographic characterization of the study population (*n* = 1304)

Parameter	Number (%)	Median (IQR)
Age	-	72.0 (64.0–78.0)
Gender	Male: 997 (76.5) Female: 307 (23.5)	-
ASA classification	-	3.0 (2.0–3.0)
Body Mass Index	-	25.4 (22.8–28.6)
Preoperative hydronephrosis	No: 1046 (80.2) Yes: 258 (19.8)	-
Neoadjuvant therapy	No: 870 (66.7) Yes: 433 (33.3)	-
Preoperative instillation therapy	No: 1039 (79.7) Yes: 265 (20.3)	-
Number of preoperative TUR-BT	-	1.0 (1.0–2.0)
Pathological diagnosis	Urothelial: 1195 (91.6) Small-cell/neuroendocrine: 22 (1.7) Squamous cell: 21 (1.6) Adenocarcinoma: 15 (1.2) Other: 51 (3.9)	-
Number of evaluated lymph nodes	-	20.0 (13.0–28.0)
Number of positive lymph nodes	-	0.0 (0.0–0.0)
Surgical resection	R0: 1180 (90.5) R1: 110 (8.4) R2: 14 (1.1)	-
Duration of Surgery in minutes	-	331.5 (265.0–390.8)
Intensive care unit	No: 644 (49.4) Yes: 660 (50.6)	-
Inpatient treatment in days	-	16.0 (12.0–20.0)
Intraoperative bleeding	No: 1295 (97.9) Yes: 28 (2.1)	-
Blood loss in ml	-	400.0 (200.0-700.0)
Blood transfusion	No: 1133 (86.9) Yes: 171 (13.1)	-
Number of packed red blood cells	-	0.0 (0.0–0.0)
Surgical technique	Open: 838 (64.3) Robot-assisted: 431 (33.1) Laparoscopic: 6 (0.5) Converted to open: 29 (2.2)	-
Urinary diversion	Ileal conduit: 881 (67.6) Ileal neobladder: 247 (18.9) Pouch: 42 (3.2) UCNS: 134 (10.3)	-
Nerve sparing	No: 1004 (77.0) Unilateral: 81 (6.2) Bilateral: 219 (16.8)	-

ASA = American Society of anesthesiologists; IQR = interquartile range; UCNS: Ureterocutaneostomy

More than one quarter (*n* = 344; 26.4%) were teaching surgeries. There were no significant differences in age (*p* = 0.175), gender (*p* = 0.658), ASA classification (*p* = 0.213) and BMI (*p* = 0.273) between teaching versus non-teaching surgeries. Regarding the operating parameters, there was a significant difference between teaching and non-teaching RC in surgical technique (significantly more open approaches for teaching versus non-teaching surgeries: 80.8% versus 58.3%; *p* < 0.001) and performed nerve-sparing (significantly less nerve sparing in non-teaching compared to teaching surgeries: 21.3% versus 27.9%; *p* = 0.008), but none in the type of urinary diversion (*p* = 0.617).

### Differences in operating results – primary study aim

There were no significant differences between teaching and non-teaching operative results in terms of surgical resection (teaching R1 status 9.9% versus non-teaching 7.9%; *p* = 0.295), duration of inpatient treatment (teaching median 15.0 days versus non-teaching 16.0 days; *p* = 0.394), infection (teaching 9.8% versus non-teaching 14.2%; *p* = 0.023), wound healing disorders (teaching 2.9% versus non-teaching 2.0%; *p* = 0.484), duration of surgery (teaching median 330.0 min versus non-teaching 332.0 min; *p* = 0.365), intraoperative bleeding (teaching 0.9% versus non-teaching 0.6%; *p* = 0.635) and intraoperative blood loss (teaching median 400ml versus non-teaching 400ml; p = 0.074). Further details are also given in the Supplementary.

However, in terms of the number of resected lymph nodes, blood transfusion rate, number of transfused packed red blood cells, intraoperative complications as well as the highest grade of complication during inpatient treatment teaching RC showed less favorable results (Table 2).

**Table 2:** Comparison of teaching and non-teaching radical cystectomy, significant differences (*n* = 1304)

Parameter		Teaching	Non-teaching	*p* value
Number of evaluated lymph nodes		Median 18.0	Median 20.0	< 0.001
Intensive care unit		Yes: 39.2%	Yes: 54.9%	< 0.001
Blood transfusion		Yes: 18.0%	Yes: 11.4%	0.002
Number of packed red blood cells		Mean: 0.34	Mean: 0.21	0.002
Intraoperative complications		Yes: 9.3%	Yes: 2.4%	< 0.001
Highest grade of complication during inpatient treatment (Clavien-Dindo classification)		Mild (I + II): 48.5% Serious (III, IV, V): 23.8%	Mild (I + II): 43.5% Serious (III, IV, V): 19.5%	0.002

### Subgroup analysis for robot-assisted radical cystectomy – secondary study aim

There were 431 RARC surgeries performed in this cohort with a median patient age 71.0 year (IQR 63.0–77.0). Of those patients 334 (77.5%) were male and 97 (22.5%) were female. Only 61 RARCs (14.2%) were teaching surgeries. There were no significant differences in age (*p* = 0.848), gender (*p* = 0.367), ASA classification (*p* = 0.074) and BMI (*p* = 0.931) between teaching versus non-teaching RARC. A detailed characterization is given in the Supplementary material.

Regarding the operating parameters, there was a significant difference between teaching and non-teaching surgery in terms of urinary diversion (significantly more continent urinary diversion in non-teaching compared to teaching RARC: 23.0% versus 18.0%; *p* = 0.007) and nerve-sparing performed (significantly more nerve sparing in teaching compared to non-teaching surgeries: 37.7% versus 24.1%; *p* = 0.008; *p* = 0.023).

In terms of operative results, there were no significant differences between teaching and non-teaching RARC for surgical resection (teaching 11.5% versus non-teaching 5.9%; R1 status; *p* = 0.068), intensive care unit (teaching 41.0% versus non-teaching 52.4%; *p* = 0.094), highest grade of complication during inpatient treatment (Severe complication in teaching 13.1% versus non-teaching 21.4%; *p* = 0.232), duration of inpatient treatment (teaching median 12.0 days versus non-teaching 14.0 days; *p* = 0.080), infection (teaching 13.1% versus non-teaching 11.9%; *p* = 0.786), wound healing disorders (teaching 1.6% versus non-teaching 1.4%; *p* = 0.859), duration of surgery (teaching median 360.0 min versus non-teaching 352.o minutes; *p* = 0.625), intraoperative bleeding (teaching 1.6% versus non-teaching 0.3%; *p* = 0.145), intraoperative blood loss (teaching median 250 ml versus non-teaching 250 ml; *p* = 0.388), blood transfusion (teaching 1.6% versus non-teaching 3.0%; *p* = 0.558), number of transfused packed red blood cells (teaching median 0.0; *p* = 0.434) and intraoperative complication (teaching 13.1% versus non-teaching 7.6%; *p* = 0.992). Interestingly, there was a significant difference in number of evaluated lymph nodes with a median number of nodes for teaching RARC of 16.0 (IQR 8.5–23.0) and 21.0 (IQR 15.0–29.0) for non-teaching surgery (*p* < 0.001). Additional details are presented in the Supplementary material.

## Discussion

To the best of our knowledge, we provide the first prospective, multicenter data answering the question, if teaching RC for BC is safe, which is of special interest since RC is considered one of the most complex urological surgeries. Unfortunately, most studies have focused only on learning curves or surgeons´ volume [4, 6, 10]. According to our data, teaching RC seems to be safe in expert centers, including RARC, but some attention must be paid to some aspects of the procedure and in education, as will be discussed below.

Firstly, we would like to discuss ORC. In our cohort, ORC is more often taught than RARC. Basic patient data were not significantly different between teaching and non-teaching surgeries. Interestingly, there was a significant higher blood transfusion rate in the teaching group despite the fact that there was no difference in intraoperative bleeding or intraoperative blood loss, so the explanation is not quite obvious. In our opinion, it could be due to selection bias: The patients are less likely at the intensive care unit and then you might give a transfusion more likely to a patient who underwent a teaching surgery. This fact needs to be addressed in further evaluations. However, teaching RCs have a higher rate on complications, especially severe ones, although the number still lies in the range of expectation for RC [3]. Therefore, it is essential to have a stringent pre- and postoperative patient management with closer monitoring for teaching RC. Such management programs should be developed and continuously evaluated to improve surgical education while ensuring patient safety. Interestingly, there are more cases in intensive care unit in the non-teaching group. The explanation is also not quite obvious, but it might be also due to selection bias since more complex cases or multimorbid patients are more likely to be operated by experienced and fast surgeons.

Secondly, we would like to discuss RARC in a more detailed way, since in the near future RARC is likely to become the gold standard for the operative treatment of muscle-invasive BC. Published data show a benefit of RARC over ORC in terms of blood loss, hospitalization time, blood transfusion or thrombo-embolic events [13, 14, 15]. Additionally, due to demographic development urologist will see more and more geriatric patients who might benefit from minimally-invasive approaches. Thus, more next generation, minimal-invasive orientated urologists will be needed. Consequently, RARC must be taught more intensively in the future. Fortunately, this seems to be safe according to our data: operative results in our cohort were similar between the two groups. However, there was a smaller number of resected lymph nodes in the teaching cohort. Therefore, special attention should be given to the precise lymph node dissection during teaching. This calls for standardized education programs in lymph node dissection in RARC. Unfortunately, a lot of recent studies call for further development and optimizing of RARC, especially for intracorporal urinary diversion [13, 14, 15, 16], but teaching aspects are seldom addressed. To the best of our knowledge, no RARC curriculum has been formally validated. Only a proposal for a curriculum for RARC with ileal conduit in male patients has been published [14, 17]. However, as mentioned above, teaching RARC is safe and due to the demographic development, we do need more teaching for this type of surgery. Consequently, it is of utmost importance to establish comprehensive curricula and to further validate and to continuously improve them. Further research in surgical education is absolutely essential to ensure patient safety and quality of care – by the way, not only in RARC. At least in our opinion, it is not enough to only describe learning curves.

In the context of education another new aspect needs to be discussed. Generation Z (year 1996–2015) will influence our department and education structure in the near future. In addition, there is a growing number of publications about Generation Z students that show that they have different needs and expectations than previous generations. Above all, these students are heavily reliant on information technology and prefer to learn and work independently at their own pace [18, 19]. This can have advantages, but also disadvantages for both collaboration and the learning environment. Therefore, surgical education programs will be different in the future, e.g. integrating digital learning tools or artificial intelligence. These special needs must also be addressed in further research.

We must assume that our study is not without limitations, e.g. we cannot exclude that some centers might have used their own definitions for complications leading to a selection bias as well as a reporting bias. Moreover, a more detailed analysis of urinary diversion, especially in RARC, would give more precise implications for the development of RARC curricula. In our opinion, further prospective educational studies should focus on this detail. In contrast, this is the first study to address patients´ surgical safety during teaching in a large, multi-center scale. However, the main limitation of this pilot analysis is that there is no teaching aspects included in the data base. Additionally, the classification of “teaching operations” relied on registry coding and did not capture the level of trainee involvement, supervision details or surgical volume, which may vary across centers. Therefore, the teaching approaches might be very different in Swiss HSM hospitals, but this fact also calls for action to develop a standardized curriculum to teach RC, especially RARC.

Despite these limitations, the study also benefits from some important strengths. The registry is mandatory in the Swiss HSM program, ensuring comprehensive case capture and a high patient volume. Diagnostic and therapeutic approaches across participating institutions closely reflect contemporary clinical standards. In addition, routine audits contribute to high data quality. Together, these factors support the robustness and reliability of this study.

In summary, teaching RC, especially RARC, seems to be safe according to our pilot analysis. There is, however, an urgent need for the development of a RARC curriculum for surgical education and its further validation. Special attention should be given to lymph node dissection, the special needs of educating Generation Z and new aspects, which have arisen, like perioperative immunotherapy [5].

## Supplementary Information

Below is the link to the electronic supplementary material.

Supplementary Material 1

## Data Availability

All data are available from the corresponding author by request.
