# Correction: How safe is teaching radical cystectomy?

**DOI:** 10.1007/s00345-026-06470-6

**Published:** 2026-06-15

**Authors:** Matteo Scherrer, Lujza Brunaiova, Marc Furrer, Hubert John, Julien Schwartz, Ilaria Lucca, Philippe Sèbe, Agostino Mattei, Daniel Engeler, Räto T. Strebel, Stephen Wyler, Thomas R. W. Hermann, Daniel Nguyen, Michael Müntener, Beat Roth, Laila Schneidewind

**Affiliations:** 1 Department of Urology, University Hospital Bern, Inselspital, University of Bern, Wilhelm-Fabry-Haus, Freiburgstr. 37, CH-3010 Bern, Switzerland; 2 Department of Urology, Canton Hospital Winterthur, Winterthur, Switzerland; 3 Department of Urology, Hospital Cecile Lausanne, Lausanne, Switzerland; 4 Department of Urology, CHUV Lausanne, Lausanne, Switzerland; 5 Department of Urology, University Hospital Genf, Genf, Switzerland; 6 Department of Urology, University and Teaching Hospital of Lucerne, University of Lucerne, Lucerne, Switzerland; 7 Department of Urology, Canton Hospital St. Gallen, St. Gallen, Switzerland; 8 Department of Urology, Canton Hospital St. Graubünden, Chur, Switzerland; 9 Department of Urology, Canton Hospital St. Aarau, Aarau, Switzerland; 10 Department of Urology, Canton Hospital Frauenfeld, Frauenfeld, Switzerland; 11 Division of Urology, Department of Surgical Sciences, Stellenbosch University, Stellenbosch, Western Cape South Africa; 12 Hannover Medical School, Hannover, Germany; 13 Department of Urology, Hospital Neuchâtel – Pourtalès, Neuchatel, Switzerland; 14 Department of Urology, Hospital Zürich Triemli, Zürich, Switzerland

**Correction: World Journal of Urology (2026) 44:133**

10.1007/s00345-025-06173-4

In the original version of the article, the following author name has been incorrectly published.

Incorrect name: Thomas Hermann

Correct Name: Thomas RW Herrmann

The original article has been corrected.

