# Supplementary material for: How safe is teaching radical cystectomy? Results from the prospective Swiss society of urology database
Source: World J Urol. 2026 Jan 28;44(1):133. doi: 10.1007/s00345-025-06173-4 (PMC12852155; doi:10.1007/s00345-025-06173-4)
Supplement: Supplementary file 1 — Supplementary Material 1 [file 345_2025_6173_MOESM1_ESM.docx]

**Supplementary**

**Extracted variables**

| Variable | Explanation |
| --- | --- |
| Age | Continuous (in years) |
| Gender | Categorial (male/female) |
| BMI | Continuous (metric) |
| Hydronephrosis | Categorial (yes/no) |
| Neoadjuvant therapy | Categorial (yes/no) |
| Pre-operative bladder instillation | Categorial (yes/no) |
| TUR-BT | Categorial (yes/no) |
| Number of pre-operative TUR-BTs | Continuous (number) |
| Histological type of cancer | Categorial (urothelial/squamous cell/small cell/neuroendocrine/other) |
| Number of evaluated lymph nodes | Continuous (number) |
| Number of positive lymph nodes | Continuous (number) |
| Surgical resection, R status | Categorial (R0/R1/R2) |
| Intensive care unit | Categorial (yes/no) |
| Intraoperative complications | Categorial (yes/no) |
| Highest grade of intraoperative complication | Categorial (no complication/mild/serious) |
| Highest grade of complication during inpatient treatment | Categorial (no complication/mild/serious) |
| Duration of inpatient treatment | Continuous (in days) |
| Charlson Comorbidity Index (CCI) | Continuous (metric) |
| Infection | Categorial (yes/no) |
| Wound healing disorder | Categorial (yes/no) |
| Bleeding | Categorial (yes/no) |
| ASA classification | Categorial (I/II/III/IV/V) |
| Classification of surgeon | Categorial (Head of department, senior consultant/consultant/resident/attending physician) |
| Teaching | Categorial (yes/no) |
| Duration of surgery | Continuous (in minutes) |
| Blood loss | Continuous (in ml) |
| Blood transfusion | Categorial (yes/no) |
| Number of transfused packed red blood cells | Continuous (number) |
| Technique of surgery | Categorial (open/robot/laparoscopic/converted) |
| Type of urinary diversion | Categorial (ileal conduit/ileal neobladder/pouch/ UCNS/other) |
| Technique of urinary diversion | Categorial (open/robot/laparoscopic/converted) |
| Type of used intestinal segment | Categorial (none/ileal/ileal-caecal/sigmoidal) |
| Nerve sparing | Categorial (none/unilateral/bilateral) |
| Seminal vessel sparing | Categorial (yes/no) |

**Table: Demographic characterization between teaching and non-teaching surgeries (n=1304)**

| Parameter | Teaching  (Number/percentage or  Median/IQR)  N=344 | Non-Teaching  Number/percentage or  Median/IQR)  N=960 | p value |
| --- | --- | --- | --- |
| Age | 70.0 (63.0 – 78.0) | 72.0 (64.0 – 77.8) | 0.175 |
| Gender | Male: 266 (77.3)  Female: 78 (22.7) | Male: 731 (66.1)  Female: 229 (23.9) | 0.658 |
| ASA classification | 3.0 (2.0 – 3.0) | 3.0 (2.0 – 3.0) | 0.213 |
| Body Mass Index | 25.1 (23.0 – 28.7) | 25.1 (22.5 – 28.5) | 0.273 |
| Preoperative hydronephrosis | No: 251 (73.0)  Yes: 93 (27.0) | No: 795 (82.8)  Yes: 165 (17.2) | - |
| Neoadjuvant therapy | No: 230 (66.9)  Yes: 114 (33.1) | No: 640 (66.7)  Yes: 320 (33.3) | - |
| Preoperative instillation therapy | No: 283 (82.3)  Yes: 61 (17.7) | No: 756 (78.8)  Yes: 204 (21.3) | - |
| Number of preoperative TUR-BT | 1.0 (1.0 – 2.0) | 1.0 (1.0 – 2.0) | 0.162 |
| Pathological diagnosis | Urothelial: 318 (92.4)  Small-cell/neuroendocrine: 5 (1.5)  Squamous cell: 9 (2.6)  Adenocarcinoma: 2 (0.6)  Other: 10 (2.9) | Urothelial: 877 (91.4)  Small-cell/neuroendocrine: 17 (1.8)  Squamous cell: 12 (1.3)  Adenocarcinoma: 13 (1.4)  Other: 41 (4.3) | - |
| Number of evaluated lymph nodes | 18.0 (11.0 – 25.0) | 20.0 (14.0 – 29.0) | <0.001 |
| Number of positive lymph nodes | 0.0 (0.0 – 0.0) | 0.0 (0.0 – 0.0) | 0.797 |
| Surgical resection | R0: 306 (89.0)  R1: 34 (9.9)  R2: 4 (1.2) | R0: 871 (90.7)  R1: 76 (7.9)  R2: 3 (0.4) | - |
| Duration of Surgery in minutes | 330.0 (270.0 – 394.5) | 332.0 (257.3 – 390.0) | 0.365 |
| Intensive care unit | No: 209 (60.8)  Yes: 135 (39.2) | No: 433 (45.1)  Yes: 527 (54.9) | <0.001 |
| Inpatient treatment in days | 15.0 (12.0 – 21.0) | 16.0 (12.0 – 20.0) | 0.394 |
| Intraoperative bleeding | No: 341 (99.1)  Yes: 3 (0.9) | No: 954 (99.4)  Yes: 6 (0.6) | 0.635 |
| Blood loss in ml | 400.0 (250.0 – 700.0) | 400.0 (200.0 – 677.5) | 0.074 |
| Blood transfusion | No: 282 (82.0)  Yes: 62 (18.0) | No: 851 (88.6)  Yes: 109 (11.4) | 0.002 |
| Number of packed red blood cells | 0.0 (0.0 – 0.0) | 0.0 (0.0 – 0.0) | - |
| Surgical technique | Open: 278 (80.8)  Robot-assisted: 61 (17.7)  Laparoscopic: 0 (0)  Converted to open: % (1.5) | Open: 560 (58.3)  Robot-assisted: 370 (38.5)  Laparoscopic: 6 (0.6)  Converted to open: 24 (2.5) | - |
| Urinary diversion | Ileal conduit: 229 (66.6)  Ileal neobladder: 66 (19.2)  Pouch: 12 (3.5)  UCNS: 37 (10.7) | Ileal conduit: 652 (67.9)  Ileal neobladder: 181 (18.9)  Pouch: 30 (3.1)  UCNS: 97 (10.1) | - |
| Nerve sparing | No: 248 (72.1)  Unilateral: 21 (6.1)  Bilateral: 75 (21.8) | No: 756 (78.8)  Unilateral: 60 (6.3)  Bilateral: 144 (15.0) | - |

*ASA = American Society of anesthesiologists; IQR = interquartile range; UCNS: Ureterocutaneostomy*

**Table: Demographic characterization between teaching and non-teaching robot-assisted radical cystectomy (n=431)**

| Parameter | Teaching  (Number/percentage or  Median/IQR)  N=61 | Non-Teaching  Number/percentage or  Median/IQR)  N=370 | p value |
| --- | --- | --- | --- |
| Age | 69.0 (61.5 – 78.0) | 71.0 (64.0 – 77.0) | 0.848 |
| Gender | Male: 50 (82.0)  Female: 11 (18.0) | Male: 284 (76.8)  Female: 86 (23.2) | 0.367 |
| ASA classification | 3.0 (3.0 – 3.0) | 3.0 (2.0 – 3.0) | 0.074 |
| Body Mass Index | 25.5 (22.8 – 28.9) | 26.0 (22.8 – 28.6) | 0.931 |
| Preoperative hydronephrosis | No: 46 (75.4)  Yes: 15 (24.6) | No: 305 (82.4)  Yes: 65 (17.6) | - |
| Neoadjuvant therapy | No: 37 (60.7)  Yes: 24 (39.3) | No: 251 (67.8)  Yes: 119 (32.2) | - |
| Preoperative instillation therapy | No: 48 (78.7)  Yes: 13 (21.3) | No: 295 (79.7)  Yes: 75 (20.3) | - |
| Number of preoperative TUR-BT | 1.0 (1.0 – 2.0) | 1.0 (1.0 – 2.0) | 0.636 |
| Pathological diagnosis | Urothelial: 59 (96.7)  Small-cell/neuroendocrine: 0 (0.0)  Squamous cell: 2 (3.3)  Adenocarcinoma:  Other: 0 (0.0) | Urothelial: 352 (95.1)  Small-cell/neuroendocrine: 5 (1.4)  Squamous cell: 3 (0.8)  Adenocarcinoma:  Other: 10 (2.7) | - |
| Number of evaluated lymph nodes | 16.0 (8.5 – 23.0) | 21.0 (15.0 – 29.0) | <0.001 |
| Number of positive lymph nodes | 0.0 (0.0 – 0.0) | 0.0 (0.0 – 0.0) | 0.367 |
| Surgical resection | R0: 53 (86.9)  R1: 7 (11.5)  R2: 1 (1.6) | R0: 344 (93.0)  R1: 22 (5.9)  R2: 2 (0.5) | - |
| Duration of Surgery in minutes | 360.0 (261.5 – 415.0) | 352.0 (290.8 – 425.3) | 0.625 |
| Intensive care unit | No: 36 (59.0)  Yes: 25 (41.0) | No: 175 (47.3)  Yes: 194 (52.4) | 0.094 |
| Inpatient treatment in days | 12.0 (9.5 – 17.0) | 14.0 (10.0 – 18.0) | 0.080 |
| Intraoperative bleeding | No: 60 (98.4)  Yes: 1 (1.6) | No: 369 (99.7)  Yes: 1 (0.3) | 0.145 |
| Blood loss in ml | 250.0 (100.0 – 300.0) | 250.0 (150.0 – 350.0) | 0.388 |
| Blood transfusion | No: 60 (98.4)  Yes: 1 (1.6) | No: 359 (97.0)  Yes: 11 (3.0) | 0.558 |
| Number of packed red blood cells | 0.0 (0.0- 0.0) | 0.0 (0.0- 0.0) | 0.434 |
| Urinary diversion | Ileal conduit: 37 (60.7)  Ileal neobladder: 11 (18.0)  Pouch: 0 (0.0)  UCNS: 13 (21.3) | Ileal conduit: 248 (67.0)  Ileal neobladder: 72 (19.5)  Pouch: 13 (3.5)  UCNS: 37 (10.0) | - |
| Nerve sparing | No: 38 (62.3)  Unilateral: 2 (3.3)  Bilateral: 21 (34.4) | No: 281 (75.9)  Unilateral: 10 (2.7)  Bilateral: 79 (21.4) | - |

*ASA = American Society of anesthesiologists; IQR = interquartile range; UCNS: Ureterocutaneostomy*
